# Characterization of the complete plastid genome of the perennial herb *Astragalus complanatus* Bunge (Fabales: Fabaceae)

**DOI:** 10.1080/23802359.2021.1961628

**Published:** 2021-11-18

**Authors:** Jin Yang

**Affiliations:** Department of Physical Education, Xi'an University of Posts and Telecommunications, Xi'an, People’s Republic of China

**Keywords:** Bayesian inference, medicinal plant, phylogenetic tree, plastid genome

## Abstract

*Astragalus complanatus* Bunge is a perennial herb with high medicinal value, and is endemic to China. The plastid genome of *A. complanatus* was determined to be 124,213 bp in length with an A + T-biased nucleotide composition (65.7% A + T). The plastid genome harbors a total of 111 genes, including 75 protein-coding, 32 tRNA, and 4 rRNA genes. The presence of one or two introns was detected in 11 protein-coding genes and 6 tRNA genes. Phylogenetic analysis revealed that *A. complanatus* failed to cluster together with the seven congeners, implying that its phylogenetic placement may need further investigation.

*Astragalus complanatus* Bunge is a perennial herb within the family Fabaceae (order Fabales), and is widely distributed in China’s Gansu, Hebei, Henan, Jiangsu, Jilin, Ningxia, Qinghai, Shaanxi, Shanxi and Sichuan Provinces with an elevation of 1,000–1,700 m (Xu et al. 2010). It is a widely used herbal material in traditional Chinese medicine, and has been extensively used for treating liver and kidney complaints (Hu et al. 2009). Besides, many studies also indicates that it has the functions of anti-liver fibrosis, inhibiting platelet aggregation, reduction of serum lipids, anti-peroxidation of lipids, anti-inflammatory, anti-tumor, immune enhancement and protection against hepatic injury (Hu et al. 2009; Qi et al. 2011). To date, most studies of *A. complanatus* have focused upon its chemical composition (e.g. Cui et al. 1992; Li et al. 2016) and pharmacological effects (e.g. Li et al. 2005; Liu et al. 2005; Qi et al. 2011; Zhu et al. 2015). However, little is known about its genetics and genomics. The present study characterized the first plastid genome for *A. complanatus*, and further investigated its phylogenetic placement as well.

Fresh leaves were sampled from an individual of *A. complanatus* from Zhaodu Township, Dali County, Shaanxi Province, China (108°39′38ʺE, 34°19′24ʺN), stored in alcohol and transported back to the laboratory for the subsequent DNA extraction. A voucher specimen was deposited at the Herbarium of Department of Physical Education, Xi'an University of Posts and Telecommunications (http://www.xiyou.edu.cn/; Jin Yang, Email: yjlemon@sohu.com) under the accession number ACOMP-2017-11-27. Genomic DNA was isolated with the DNeasy Plant Mini Kit (Qiagen, CA, USA). High-throughput DNA sequencing was conducted on the Illumina HiSeq X Ten Sequencing System (Illumina, CA, USA), which totally yielded 51.51 M of 150-bp paired-end reads. The plastid genome was assembled using the program NOVOPlasty v4.2.1 (Dierckxsens et al. 2017) with that of *Astragalus nakaianus* (GenBank accession: KR296789) (Choi et al. 2016) as the reference sequence. Genome annotation was done in Geneious Prime 2020 (Biomatters Ltd., Auckland, New Zealand) by aligning with those of closely related taxa.

The plastid genome of *A. complanatus* was successfully recovered with an average coverage of 116.5X. It is 124,213 bp in size with a biased A + T content of 65.7%, and encodes a panel of 111 genes, including 75 protein-coding (PCG), 32 tRNA, and 4 rRNA genes. One or two introns are present in 11 PCGs (*atp*F, *clp*P, *ndh*A, *ndh*B, *pet*B, *pet*D, *rpl*2, *rpl*16, *rpo*C1, *rps*12, & *ycf*3) and six tRNA genes (*trn*A-UGC, *trn*G-UCC, *trn*I-GAU, *trn*K-UUU, *trn*L-UAA, & *trn*V-UAC). Unlike most plastid genomes of angiosperms but like those of closely related taxa (Palmer et al. 1988), the plastid genome of *A. complanatus* does not have the typical quadripartite structure consisting of a large single copy (LSC), a small single copy (SSC) and a pair of inverted repeats (IRs).

Phylogeny of 36 taxa within the tribe Galegeae (Fabales: Fabaceae) was reconstructed based on the Bayesian analysis of the dataset of concatenated plastid PCG gene sequences shared by all taxa (Figure 1). Phylogenetic analysis was performed using the program MrBayes v3.1.1 (Huelsenbeck and Ronquist 2001; Ronquist and Huelsenbeck 2003) as implemented in the program TOPALi v2.5 (Milne et al. 2009). The employed best-fit nucleotide substitution is GTR + G + I. The outgroup taxa included are three taxa from the tribe Hedysareae (Fabales: Fabaceae), including *Hedysarum petrovii* (MT120797)*, Hedysarum taipeicum* (MK426698) (She et al. 2019) and *Onobrychis viciifolia* (MT528597) (Jin et al. 2021). The relationship between the two genera *Glycyrrhiza* and *Meristotropis* may need further investigation, since taxa of either genus failed to form a distinct monophyletic group. Besides, *A. complanatus* was found to be more closely related to *Lessertia frutescens* and *Sphaerophysa salsula* rather than to the seven congeners. Our finding appears to support the placement of *A. complanatus* within another genus (Xu et al. 2010).

**Figure 1.**
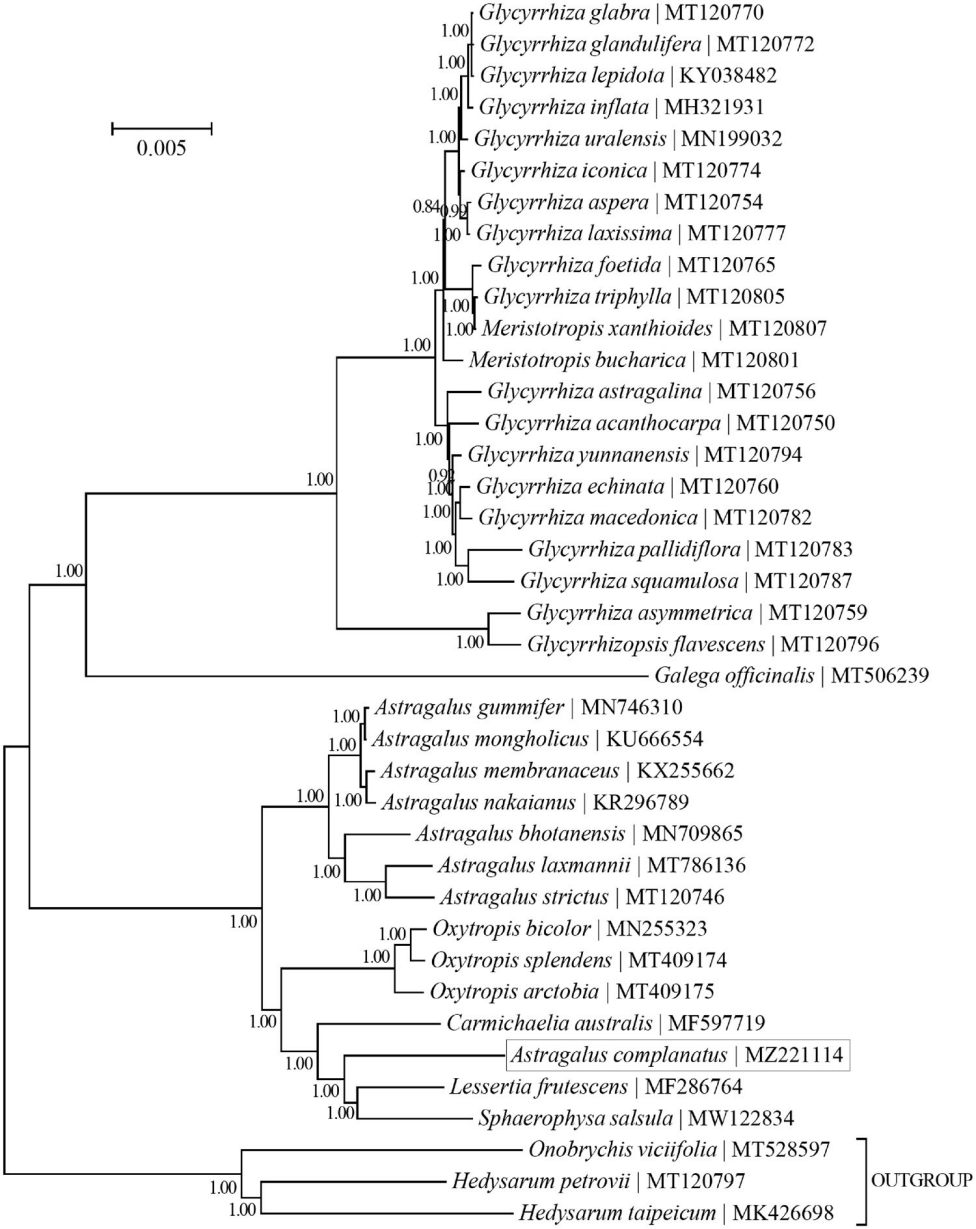
Phylogeny of 36 taxa within the tribe Galegeae (Fabales: Fabaceae) based on Bayesian analysis of the plastid protein-coding genes. The outgroup taxa included are three taxa from the tribe Hedysareae (Fabales: Fabaceae). The best-fit nucleotide substitution model is ‘GTR + G+I’. The numbers next to the nodes are posterior probabilities of the Bayesian analysis.

## Data Availability

The genome sequence data that support the findings of this study are openly available in GenBank of NCBI at [https://www.ncbi.nlm.nih.gov] under the accession number MZ221114. The associated **BioProject**, **SRA** and **Bio-Sample** numbers are PRJNA729989, SRR14547473 and SAMN19190150, respectively.
